# Rice Bran By-Product: From Valorization Strategies to Nutritional Perspectives

**DOI:** 10.3390/foods10010085

**Published:** 2021-01-04

**Authors:** Marco Spaggiari, Chiara Dall’Asta, Gianni Galaverna, María Dolores del Castillo Bilbao

**Affiliations:** 1 Department of Food and Drug, University of Parma, Parco Area delle Scienze, 17/A, 43121 Parma, Italy; marco.spaggiari1@studenti.unipr.it (M.S.); chiara.dallasta@unipr.it (C.D.); gianni.galaverna@unipr.it (G.G.); 2 Food Bioscience Group, Department of Bioactivity and Food Analysis, Instituto de Investigación en Ciencias de la Alimentación (CIAL) (CSIC-UAM), Calle Nicolás Cabrera, 9, 28049 Madrid, Spain

**Keywords:** rice bran, by-products, bioprocessing, bioactive compounds, nutritional value, rice bran arabinoxylans

## Abstract

The aim of this study is to review the innovative techniques based on bioprocessing, thermal or physical treatments which have been proposed during the last few decades to convert rice bran into a valuable food ingredient. Rice bran (*Oryza sativa*) is the main by-product of rice grain processing. It is produced in large quantities worldwide and it contains a high amount of valuable nutrients and bioactive compounds with significant health-related properties. Despite that, its application in food industry is still scarce because of its sensitivity to oxidation processes, instability and poor technological suitability. Furthermore, the health-related effects of pretreated rice bran are also presented in this review, considering the up-to-date literature focused on both in vivo and in vitro studies. Moreover, in relation to this aspect, a brief description of rice bran arabinoxylans is provided. Finally, the application of rice bran in the food industry and the main technology aspects are concisely summarized.

## 1. Introduction

**Figure 1:**
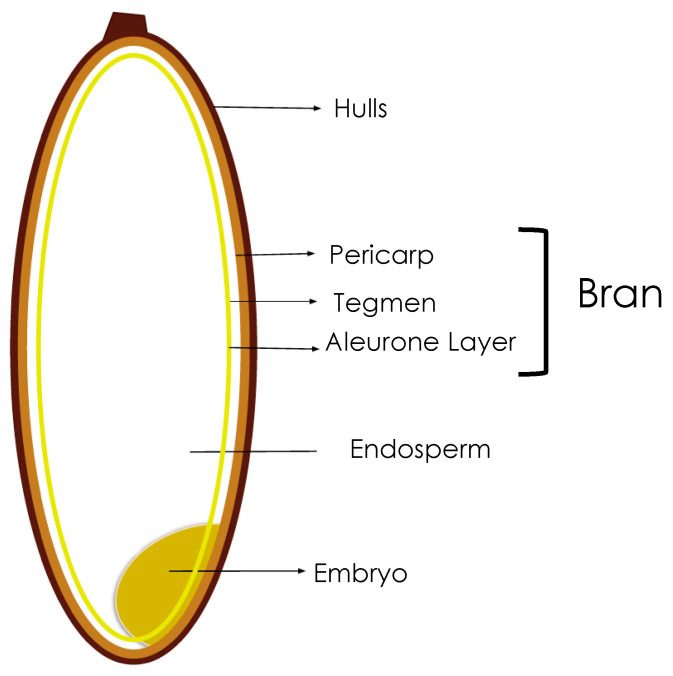
Schematic representation of rice grain, highlighting the bran fraction.

Food processing is a set of operations that permit to transform raw materials into valuable food ingredients. Cereal crops, for example, are rarely consumed as whole grains and during their transformation process a large amount of residue is produced. Rice bran (RB) is a by-product that derives from the milling of rice grain, the third most consumed cereal overworld [1]. It represents around 12% of the total kernel weight and it is composed by the external layer of the seed (i.e., pericarp, tegmen, and aleurone layer (Figure 1)), translatable in almost 68 million tons of unmanageable material per year, worldwide [2]. Similarly to other cereal species, the kernel surrounding layers are richer in bioactive compounds, minerals, vitamins, dietary fibers, proteins and lipids than the core endosperm, which is characterized by simple carbohydrates and starch granules [3,4] (Table 1). In particular, rice bran has a not-negligible amount of lipids (15–20 g/100 g of RB), where some of the most important bioactive compounds, such as γ-oryzanol, ferulic acid, tocopherol and polyunsaturated fatty acids could be found; thus, for this reason, RB has been used for oil extraction [5]. Despite that, RB is highly sensitive to lipid oxidation due to the rapid activity of lipolytic endogenous enzymes, which means that a thermal stabilization step usually required [6]. However, the first fate of rice bran is the feed formulation industry, losing the opportunity for the recovery of its potential.

Therefore, the study of innovative recovery strategies focused on the valorization of agro-industrial by-products is now an interesting growing sector directed to an improved sustainability and dietary habits of the whole food system. In this work, the strategies for the valorization of rice bran, such as fermentation, air classification and high hydrostatic pressure, were reviewed, with a special focus on their potential modification. Then, its recent nutritional evidences, health-promoting properties and utilization as functional food ingredient will be also summarized.

## 2. Research Criteria for Scientific Papers Selection

The review was organized starting with the study of the most modern techniques used for rice bran treatment. Then, the health-promoting properties related to rice bran were assessed singularly. Finally, the relevance of rice bran as ingredient in the food industry considering its functional properties were reviewed. In this literature review Google Scholar, Web of Science and Scopus research engines were consulted. Key search terms related to rice bran by-products and co-products (i.e., “rice milling by-products”, “rice bioactive compounds”), were conjointly searched with the followings: “biotechnology”, “fermentation”, “emerging technologies”, “thermal treatment”, “physical treatment”, “nutritional value”, “health-promoting properties”, “safety” and “applications”. No data filtering (i.e., year) was applied in order to gain as much knowledge on the topic as possible. The legislation framework information was reported by the European Food Safety Authority (EFSA). Paper selection was performed after a comprehensive study of the results and their discussion as reported by the authors. The output of the latter selection process highlighted 94 original research articles.

## 3. Composition and Strategies for the Recovery of Rice Bran Potential

The smart recovery of food processing residues is an important aspect for the food industry, which always is in search of value generation. In this context, a great benefit may derive from different branches of science, such as biotechnology, chemistry or physics, applied to the food scenario. There are plenty of innovative techniques applied to produce a high-value ingredient useful for the production of sustainable products by food manufacturers. Following the introduction, rice bran general composition is reported in Table 1. Macro and microelements occurrence depends on several factors including botanical variety, environmental agronomical conditions and processing.

### 3.1. Bioprocessing of Rice Bran: Solid-State Fermentation and Enzymatic Treatment

Food bioprocessing is that term used to define an operation which combines living microorganism cells or their components to a normal procedure to obtain a different food product. The processes that utilize these technologies are summarized in Table 2. Nowadays, solid-state fermentation (SSF) and the use of specific enzymes are widely studied and applied in food industry, with the principal aim to overall improve the characteristics of the raw materials treated. The most important, from a nutritional point of view, is the fact that the microorganism metabolism or selective enzymes can disrupt the vegetable cell matrix releasing those compounds that present a bioactivity, enhancing their bioavailability in the human organism. Regarding fermentation, both fungi and bacteria can take part to this process. Obviously, the metabolic characteristics of each are different, as well as the resulting raw material modifications. For rice bran fermentation, fungi and yeasts are the mostly used microorganisms. For example, in the study of Abd Razak et al. [13], *Rhizopus oligosporus* and *Monascus purpureus* was used both alone and in combination, for the fermentation of sterilized rice bran. The study analyzed the overall antioxidant capacity (AOC) and bioactive compounds enhancement caused by such treatment. Interestingly, the two microorganisms used in co-culture resulted in a significantly higher AO and phenolic acids content in respect to the non-fermented rice bran. Similar outcomes were obtained by Schmidt et al. [14], where rice bran was fermented with *Rhizopus oryzae (CCT 1217)* over 120 h. They measured AOC with different spectrophotometric and widely used methods, phenolic acids content and the inhibition activity against peroxidase enzyme, which is mainly responsible for the lipid oxidation. The AOC increased in respect to the non-treated rice bran and phenolic compounds, such as ferulic and gallic acids, doubled their initial concentration. This fact can only be attributed to the cell-wall disrupting metabolism of microorganisms, and not to their direct biosynthesis, since fungi cannot produce such secondary metabolites. Similar results were found by Shin et al., in which after the fermentation of black rice bran using *Aspergillus awamori* and *A. oryzae*, they measured a higher soluble phenolic content and radical scavenging activity [15]. Additionally, Oliveira et al. [16] used the same fungus (but a different strain), although they focused the study aim on the lipid compounds modification. Interestingly, the total lipid content of fermented rice bran diminished up to 45% after the fermentation, but the phospholipids increased by 130%, indicating that the microorganism could metabolize such molecules. Moreover, the saturated and unsaturated fatty acids content was altered, the former decreased up to 20% and the latter increased by 5%. Probably ascribed to a combined action of oxidation processes and fungus metabolism. Ryan et al. [17], followed a different but innovative approach to characterize fermented rice bran from three different rice varieties using *Saccharomyces boulardii*, a yeast. Employing a metabolomic gas chromatography coupled to mass spectrometry (GC-MS) based technique, they could identify and quantify new bioactive metabolites (i.e., glucitol and unknown disaccharides). Afterword they assessed their bioactivity on normal human peripheral blood lymphocytes (PBL) and malignant human B-cell lymphoma cell lines, resulting in a reduction of the cells growth of the latter ones. Besides phenolic compounds, rice bran polysaccharides (RBPSs) have gained more interest due to their multiple biological benefits. In the study carried out by Liu et al. [18], they fermented a defatted rice bran water extract using the fungus *Grifola frondosa*, aiming to evaluate the composition of the oligosaccharides fraction, AOC and inhibition of nitric oxide (NO) production in macrophages cells. They reported a substantial shift of the polysaccharides molecular weight, from higher to lower, besides the increased AOC and the adjustment of the NO production compared to the non-fermented sample. Furthermore, technologic properties must be considered as equally relevant as nutritional characteristics, since they can firstly affect the food design process. Therefore, Jia et al. [19] focused their study on the effect of defatted rice bran fermentation on soluble dietary fibers, studying their capacity to bind water and oil, which significantly increased after treatment.

Enzymes with specific activities are widely spread in food industry [20]. Their capacity to improve functional and sensory features of food products is accompanied by the fact that their impact on the native composition is very low [21]. Regarding cereal-related products, enzymes are usually used to modify the principal component of that food, such proteins, lipids and carbohydrates [22]. In fact, in the study conducted by Prabhu et al. [23], they treated rice bran with cellulase and xylanase with the aim to solubilize polyphenols with the corresponding improving of the AOC. Similarly, Vallabha et al. [24] treated rice bran with alcalase and endoglucanase, resulting in an increased content of bioactive molecules with no effect on the degradation of B group vitamins after the enzymatic treatment. They also reported an increment of the short chain fatty acids (SCFAs) content in respect to the non-treated rice bran. Moreover, enzymatic processes are usually utilized in combination with other techniques such as fermentation [25], extrusion cooking [26], high hydrostatic pressure (HHP) [27] or micronization [28], leading to an increased extractability of the bioactive component, solubilization of dietary fibers and inhibition of lipid oxidation.

Although these processes might lead to an overall amelioration of the raw material, their biggest disadvantage is that they need long procedures, under specific and strictly controlled conditions to be effective and with the desirable yield. This is only achievable by long term studies performed in tight collaboration between academia and industry. In fact, there is a study published this year [29] in which the authors deeply analyzed the parameters that must be taken into account for the valorization of rice milling side streams under a circular economy view. For example, they reviewed the pre-treatment, the type of fermentation (submerged, solid and semi solid-state, saccharification) useful for the bacterial growth and the subsequent production of valuable compounds like biofuels, lactic acid or biohydrogen.

Considering these treatments, the combined action of enzymes and fermentation can achieve the best results in terms of technological and functional improvements. However, the cost of the processing at industrial scale must be accurately determined. For that reason, the treatment of rice bran using lactic acid bacteria and fungus as fermentation microorganisms seems to be the best condition for obtaining an ingredient with a pleasant organoleptic profile, good nutritional properties and excellent functionalities.

### 3.2. Thermal and Physical Treatments Applied to Rice Bran

The conscious manipulation of heat has been always used for the transformation of food products in usually, quality-improved foodstuffs. In current years, several new technologies have arisen thanks to their offered potential (Table 3). Extrusion, hot air and far infrared irradiation (FIR) are some of these. For example, Wanyo et al. [30] used hot air and FIR, alone and in combination to assess the effects on the antioxidant properties and bioactives content of rice bran and husk. They reported that the use of FIR technology conferred to rice bran a higher content of bioactive compounds, mainly phenolics, and AOC in respect to the classic hot air treatment. Unfortunately, no mention of the sensorial characteristics was present in the latter study. Furthermore, in the experiment carried out by Dang and Vasanthan [26], they performed a sequential enzyme treatment followed by extrusion, concluding that an increased total soluble pentosan content compared to the to individual or simultaneous treatments. Moreover, Fadel et al. [31], Liu et al. [32] and Dang and Vasanthan [26] applied extrusion techniques at different conditions reporting an increased extraction of arabinoxylans, improved foaming and emulsifying properties and increased soluble fiber content of the extruded bran, respectively. In summary, extrusion is highly studied science field. This is mainly due to the “gold rush” towards meat analogue manufacturing which has occurred in recent years [33]. However, rice bran, and especially rice proteins, are scarcely used as a principal ingredient, since its composition is less suitable to this transformation then other products (i.e., pea protein isolate).

Nevertheless, food science and related disciplines are continuously modernizing to study innovative techniques which may have minimum impact on both products and environment. Physical treatments are those operations based on physics principals applied for many objectives. In the case of cereal products, such rice grain, the properties of porous and fine material (i.e., flour) are the main aspects that must be taken into account. Separation, concentration and physical properties modification of specific food component are the main paths followed by researchers, achieved using dry fractionation, air classification and particle size reduction technologies. These techniques usually result in production of two or more fractions of the material treated, differing at least in granulometry (i.e., fine and coarse fractions). For example, Wang et al. [34], applied pin mill fractionation coupled to an electrostatic separation to defatted rice bran. The mainly outcome authors reported was the dietary fiber enrichment found in coarse fraction of rice bran, remarking the redistribution effect proportioned by this type of technology. Moreover, in the study proposed by Spaggiari et al. [35], authors applied micronization, air-classification and the combination of these techniques in order to obtain different rice bran fractions (fine and coarse), aiming to deeply analyze the lipid profile, with special focus on mono- (MAG) and diacylglycerols (DAG). They found that the lipid content increased significantly in fine fraction, and the MAG, DAG and triacylglycerols content increased by 1.5–5 times in the former fractions. These molecules are particularly interesting to the food industry since they have strong emulsifying potential [36], and find that a “natural source” alternative instead of the additive E-421 (mono- and di-acylglycerol of fatty acids) would be a greater competitive advantage. Nevertheless, more studies have to be conducted in order to optimize this type of treatments, considering the opportunity to combine different fraction with different characteristics, and thus different functions. Furthermore, Silventoinen et al. [37] studied the biochemical and techno-functional modification occurred after the air classification of rice bran, obtaining two different ingredients, a protein-rich ingredient and a fiber-rich Ingredient. These had different microstructural and functional properties, such as protein solubility and colloidal stability, suggesting an elevated applicability in food industry. Moreover, using the HHP technique and different pressures (100, 200 and 500 MPa), Zhu et al. [38] obtained a rice bran with higher WBC and OBC parameters and improved foaming formation and stability, although with a lower least gelation concentration, meaning that the capacity to form a stiff gel requires a lower amount of rice bran. These results stress the importance of the continuous study of food functional properties in order to achieve a higher quality and sustainable ingredients.

## 4. Rice Bran Health-Promoting Properties

**Figure 2:**
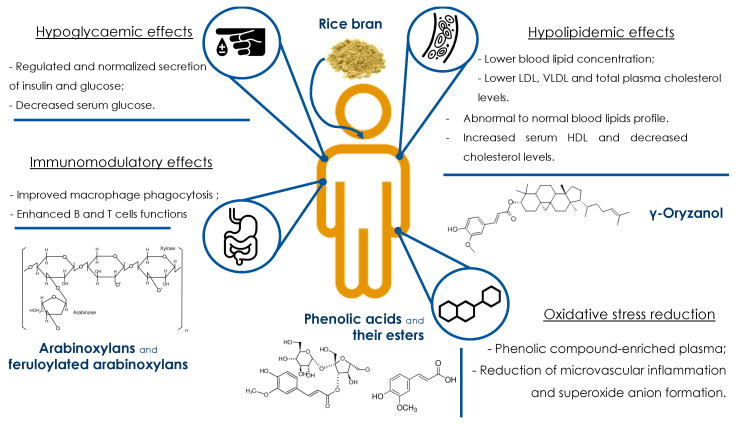
Representation of the health-promoting properties of rice bran, the compounds responsible for that characteristics and the references of the studies highlighting these capacities. Abbreviations: LDL, low density lipoprotein; HDL, high-density lipoprotein; VLDL, very low-density lipoprotein.

Our knowledge of the relationship between diet and health is becoming stronger. In relation to this, the dietary patterns of developed countries are also changing, showing a growing incidence of many diet-related disorders and diseases (cardiovascular disease (CVD), high cholesterol, diabetes, bowel inflammation, etc.). Rice bran is a by-product rich in relevant bioactive compounds which can play an important role to maintain a healthy status and thus promote a beneficial living style. However, this raw material is currently discarded notwithstanding its potential as a functional ingredient [39] or its potential chemo-preventive and immunomodulatory properties [40,41,42,43]. On the other hand, despite the scientifically proven evidence, foods and their constituents should not be considered as medical replacements but as a complementary part which contributes toward a healthy living. However, the scientific evidence-based process is at the base of the applications to specific “health or nutritional claims” [44]. This evaluation procedure is, nowadays, based on European Food Safety Authority (EFSA) opinions regarding a specific application. The health-promoting properties ascribed to rice bran or its valuable compounds are summarized in Figure 2.

### 4.1. Hypolipidemic Properties of Rice Bran

Recently, an increasing number of studies have analyzed the multiple health-related properties of a rice bran supplemented diet [43]. Besides its high nutrient and bioactive compounds content, rice bran can be administered in different ways, such as stabilized RB, fermented RB, enzymatically treated RB and oil. Each of these products differ from the raw RB, in terms of nutrients and non-nutrients compounds. For this reason, a previous profiling of the product under study is highly recommended in order to define the chemical compounds or mixture of compounds which deliver the desired effects of a previously determined diet.

Health conditions that determine an increased concentration of lipid molecules and cholesterol in blood are a branch of a disease known as hyperlipidemia. These can raise from both genetic conditions and bad dietary habits, which could finally lead to an increased risk of CVD. For this reason, nutrition is the most important variable that must be taken under control for the treatment of such ailment. From this perspective, rice bran and its oil are rich in components which can contrast the accumulation of lipids in blood stream, such as phytosterols, unsaturated fatty acids (FAs) and tocotrienols [41,45]. In fact, the oil recovered from rice bran include glycolipids, phospholipids, free fatty acid and triglycerides with healthy profile, since oleic, linoleic (mono- and polyunsaturated) and palmitic acids (saturated) are the most abundant FAs. In a recent study, Perez-Ternero et al. [46] evaluated the lipid-lowering properties of rice bran enzymatic extract (RBEE) diet supplementation in apolipoprotein E-knockout (ApoE−/−) mice. They reported a higher high-density lipoprotein (HDL) serum value and an increased cholesterol excretion. The same authors reported also other protective properties of RBEE, such as a restored endothelial function, hyperlipidemia prevention, oxidative stress, inflammation and apoptosis reduction in the aorta of ApoE−/− mice [47,48]. Revilla et al. [49] also used RBEE as diet supplementation in male Wistar rats, which resulted in an increased HDL and lower blood cholesterol concentrations in plasma. Besides, Wilson et al. [50] settled up a comparative study in which they evaluated the potential of trans-ferulic acid, γ-oryzanol and rice bran oil supplementation to lower the cholesterol concentration in primates. Among the groups studied, the one fed with γ-oryzanol showed the lower levels of low and very low-density lipoprotein (LDL and VLDL) and plasma cholesterol. Similar outcomes were reported by Ausman et al. [51] and Ha et al., [52], where they fed hypercholesterolemic hamsters and Male Sprague–Dawley rats, respectively, with different amount of rice bran oil. Moreover, Accinni et al. [53] studied the effects of various dietary supplementation, including γ-oryzanol, on the lipid profile of dyslipidemic subjects. Interestingly, results reported by the latter study indicate that the subjects which followed a supplementation diet with γ-oryzanol and niacin recovered a normal lipid blood pattern, better than the other food supplementation studied.

### 4.2. Hypoglycaemic Properties of Rice Bran

The presence of high level of glucose in blood system is a disturb that, nowadays, is extremely widespread in developed country populations, which is mainly associated with bad diet habits and thus could lead to a harmful health status, such as diabetes and hyperglycemia. In this way, vegetable origin food products, such as rice bran, that contain low free sugar and high dietary fiber content can contribute to maintain a lower glycemic index and help to the illness prevention. For example, Son et al. [54] studied the regulation effects of γ-oryzanol on the insulin secretion and glucose concentration in plasma, supplemented to male C57BL/6N mice. The same results were reported in the study of Somsuvra and Ghatak [55], in which adult Wistar rats with a γ-oryzanol supplemented diet had the lower serum glucose content. Moreover, Qureshi et al. [56] expanded the study to humans’ volunteers with Type I and II diabetes mellitus. Rice bran was administered as stabilized and as water extract, leading to an increased insulin serum level, decreased glucose and glycosylated hemoglobin concentration.

### 4.3. Rice Bran Arabinoxylans: Immunomodulatory Properties

Recently, the saccharide component of foods has been receiving more attention. The main reason of this is the fact that carbohydrates have never been studied in depth, due to major analytical issues [57]. Despite this, many biological activities have been attributed to molecules including arabinoxylans which have low and high molecular weight. As is widely known, carbohydrates are major components of cereals; in fact, they have the primary function of seed energy storage. In detail, arabinoxylans are composed by xylan backbone (β-(1,4)-D-xylopyranose) linked to α-L-arabinofuranosyl substitutions, which differs among cereal species, conferring different arrangements and thus different functions [58]. In this context, it is crucial to accurately study the chemical composition and potential functionality correlated to arabinoxylans [59]. In fact, a huge effort in terms of research is being made to study the rice bran arabinoxylans-immunomodulatory relationship, which is capable of enhancing the activity of the innate and adaptative responses. These mechanisms are responsible for activating the B-lymphocytes production, our first line of defence from foreign molecules [60,61]. In this way, rice bran is usually enzymatically treated, in order to break high molecular weight arabinoxylans into smaller pieces, in the range of 30–50 Da, producing a product commercially called BioBran or MGN-3 [62]. These fraction appear to act as an enhancer of natural killer cell, macrophage phagocytosis, B and T cells functions [39]. Ghoneum et al. studied these effects in depth [63,64,65,66,67,68,69]. Furthermore, Choi et al. [70] reported similar outcomes after supplementation of the soluble arabinoxylans fraction of wheat bran in mice diet. A proposed theory of the mechanism of action has been proposed [71], stating that the complexity of the structure of the heteropolysaccharides such as galactan, arabinan and β-1,3:1,4- glucan is the basis of the immunomodulatory actions of rice bran arabinoxylans. Other properties (Figure 2) of rice bran arabinoxylans were reported by Salama et al. [72], where the MGN-3 supplementation in patient with chronic hepatitis C virus suppressed the level of viremia. Otherwise, Zheng et al. [73,74] reported protective effects against acute liver injury. In addition, Wang et al. [75] showed an improved anticomplementary activity of DRB under in vitro conditions. Numerous original articles have been published recently which mention the MGN-3 commercial product, although its discussion is not the main focus of these reviews.

### 4.4. Other Health-Promoting Properties of Rice Bran

Since a wide range of bioactive molecules are present in rice bran, the effects on the oxidative stress are also currently object of study. Justo et al. [76] reported a reduction in microvascular inflammation status in obese Zucker rats which follow a diet supplemented with RBEE. Moreover, Perez-Ternero et al. [77] also studied the supplementation of RBEE in Wistar rats’ diet, showing an inactivated superoxide production caused by an increased content of phenolic compounds in blood. Other research has been made focusing on the multiple effects of rice bran supplemented under various forms. For example, fermented brown rice bran using *Aspergillus oryzae*, which induced apoptosis in human acute lymphoblastic leukemia cells [78], or the anti-stress and antifatigue effects delivered by *Saccharomyces cerevisiae* fermented rice bran [79], or otherwise, the improvement of the health condition of mice fed with a high-fat diet including rice bran extract [80]. Moreover, a dietary fibre-rich diet is known to be able to modulate the metabolites produced by human colon microbiota. As reported by Zhang et al. [81], after in vitro gastrointestinal digestion of isolated rice bran dietary fibre, a great probiotic effect on colonic microorganism associated with diabetes was found.

## 5. Rice Bran Application in Food Industry

The most important step for the recovery of food by-product is the incorporation of such raw material in a classic or novel food product formulation. The primary objective is to add value to the final product, both enhancing nutritional properties or sensorial characteristics, such as color, taste, smell and texture. The latter functions are also important, forming the labelling point of view, since the lack of additives, which are replaced by the recovered material, can lead to the so-called “clean label” status. Rice bran has also another advantage, represented by its suitability for gluten-free product manufacturing. These products usually are poor in term of sensorial quality, but have a high value in market. Moreover, functional properties are another important aspect to be considered for the inclusion of these by-products in a food formulation. These characteristics are reported and summarized in Table 4. RB is a plant material; hence, dietary fibers are the predominant substances composing this product. The latter molecules can contribute to the overall functionality of rice bran, considering its water (WBC) and oil binding (OBC) capacities. Additionally, the protein fraction present in RB (12–16%, [2]) offers potential jellifying, emulsifying and foaming stabilization activities. As stated by Capellini et al., the techno-functional properties of rice bran are interesting for the food industry and they can be improved using specific alcoholic solvents [82]. Moreover, Shi-Wen. et al. [83], reported relevant results regarding the functional properties of rice bran stabilized using different treatments such as microwave and dry heating, with the latter slightly better than the former in relation to the WBC, OBC, emulsifying properties and long-term stability.

**Figure 3:**
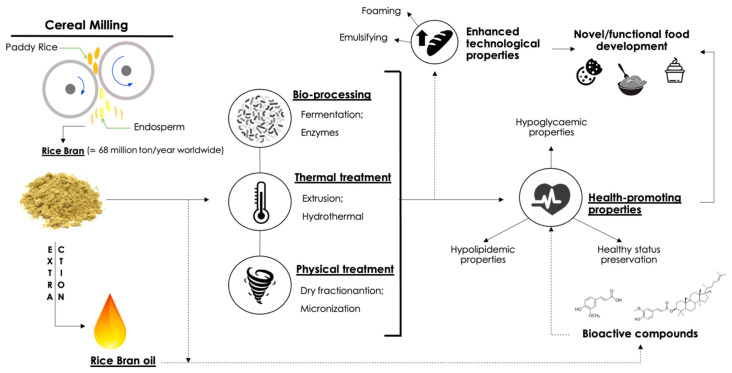
Rice bran production, the treatments applied for its improvement and the nutritional evidences for the formulation of novel or functional food ingredients.

In fact, it is highly recommendable to stabilize rice bran [85] or to extract the oil before its use as an ingredient, considering its sensibility to oxidation and the subsequent off-flavor formation. This can be easily achieved through the application of thermal and non-thermal techniques, as reported in Section 3.2. Sairam et al. [86] analyzed the addition of different concentration of defatted rice bran (DRB) in bread, aiming to improve the nutritional profile of bread. Total dietary fibres, AOC and the shelf life of bread increased with no repercussions on the sensorial properties. In relation to this, Al-Okbi et al. [87] formulated corn flakes and tortilla chips adding different amounts of rice bran to the original recipe, noticing an organoleptic and rheological amelioration of the final product when the protein content decreased. Premakumari et al. [88] also tried to develop high fiber content ready mixes by replacing the classical cereal flours with a different amount of previously stabilized RB, evaluating their overall acceptability through 20 semi-trained panel members. The main outcome showed that a replacement of at least 25% did not affect the quality of the standard recipe. Moreover, Younas et al. [89] developed cookies using both heat and acid-stabilized RB, optimizing the recipe with a 10% RB substitution. Since RB oil is considered a high added value product (Figure 3), the RB solid exhaust can still be used as a low-fat content ingredient. For example, Charunuch et al. [90] utilized DRB at a different rate for the preparation of extruded breakfast cereal. Meanwhile Alfaro et al. [91] attempted to produce an oil-in-water emulsion using purple and brown rice bran oils, achieving acceptable stability and oxidative protection. However, the use of pretreated rice bran by means of bioprocessing, thermal and physical treatments in food preparation is still scarce, possibly due to the lack of industrial-scale or pilot-scale studies. Moreover, the protein present in rice can be isolated in order to produce ingredients with high protein content, and thus with high functionalities. Regarding this, Zang et al., [92] attempted to improve the emulsification properties of enzymatically-treated rice bran protein, obtaining a more stable emulsion. Nowadays, the topic of plant protein is one which is gaining increasing interest. Therefore, the easy accessibility of rice bran protein represents a relevant resource for the food industry. The proteins recovered from rice bran have specific functionalities, bearing in mind that they are considered hypoallergenic. For this reason, this field is an open door for the food industry, considering that these substances can offer functional and bioactive properties at the same time [93].

### Rice Bran as a Commercial Ingredient

**Figure 4:**
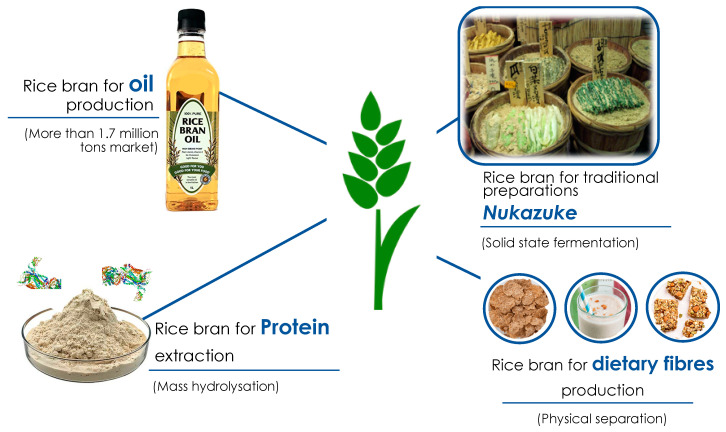
Representation of the products already present in the market or produced from rice bran by-product.

Cereals and their correlated products have always been strong cultural drivers and incubators. Besides, they provide nutrients and energy, constituting the main part of the human diet. Rice grains are typically widespread in Asian and Eastern countries in general, where they are capable of fully exploiting this product. For example, in Japan, there is a special preparation made of fermented rice bran and called “Nukazuke” (Figure 4). Different vegetables are left in this fermented biomass in order to transfer several aromas, tastes and flavors created by the microorganism consortium developed in the rice bran bed [94]; it is a simple pickling. On the contrary, rice in occidental culture is used in different ways, and rice bran has been mostly utilized as a dietary fiber enhancer in industrial food preparations. For example, in baked products such as biscuit [2], leavened pan bread [26] or cakes, the addition of 10 to 20% of stabilized rice bran delivers technological functionality, better nutritional profile and a pleasant texture, without affecting the organoleptic properties compared to the classic recipe. These aspects are extremely important since they can attract the attention of the food industry for the development of new business driven by innovative technologies. In addition, as already mentioned, the oil extracted from rice bran has a high value, although this process might produce “by-by-products”, contrasting the idea of thee circular economy and side streams exploitation. However, rice bran oil contains different bioactive compound pro-vitamins, lower saturated fatty acids and polyunsaturated fatty acids, which can be considered as a good nutritional profile. Moreover, the organoleptic characteristics are mild and, if stabilized well, it is a very stable fat [43,52]. The legislation framework is another important aspect when a food product or ingredient comes onto the market. In this case, rice bran is considered as a non-novel food, which is significant for this product. In fact, companies which produce rice bran and other food ingredients are already present and active in the European market (https://www.ricebrantech.com/specialty-ingredients/). They can integrate the entire rice food chain for the recovery of all rice processing side streams.

## 6. Conclusions and Future Perspective

Rice bran represents a massive industrial by-product with a relevant “hide” potential value. Its compositions, in terms of nutritional features, comprehend a wide range of bioactive compounds which have been studied in recent years. Furthermore, with the aid of the most advanced food-related technologies, the quality parameters of RB can be highly improved, making it a valuable and sustainable resource for healthy diet promotion. In fact, considerable efforts have been made to include this ingredient in widely consumed food products, mainly baked goods. Then, several health-promoting properties suggested the importance of including RB, in its various forms, in the diet. However, the mechanism of action underlying its positive effects should be studied more in depth. In this way, it is important to stress the fact that foods and their components must not be intended as the main instruments with which to struggle with illness. Finally, different types of innovative food products can also be studied, such as novel beverages or dairy products with high nutritional and sensory qualities, helping to achieve a more sustainable food system.

## Figures and Tables

**Table 1:** Mean proximate composition of rice bran and the content of its main bioactive compounds.

**Compound**	**mg/100 g**	**References**
Carbohydrates	33–42	[7,8,9,10,11,12]
Proteins	11–16	
Fats	12–20	
Saturated fats	15–20	
Unsaturated fats	75–80	
Dietary fibres (DF)	15–30	
Insoluble DF	13–26	
Soluble DF	1–2.25	
Ash	8–12	
**Bioactive Compounds**	**mg/g**	
Phenolic acids *	800–1243	
Tocopherols	0.35–0.77	
γ-oryzanol	0.56–1.08	

* as total ferulic acid, the most abundant phenolic acid found in cereal grains (soluble and insoluble forms).

**Table 2:** Innovative strategies based on bioprocesses applied to rice bran by-product and the novel effects achieved by their application.

Bioprocessing	Effects	References
Solid state fermentation (SSF)	Increased soluble phenolic acids content; Increased AOC; Increased the inhibition of tyrosinase activity;	[13,14,15]
	Decreased saturated fatty acids content; Increased inhibition of peroxidase enzyme; Increased phospholipids content;	[16]
	Reduction of the polymerization degree of polysaccharides; Increased solubilization of soluble dietary fibres; Potential anti-inflammatory properties; Higher WHC, OHC, WS and CAC;	[18,19]
Enzymatic treatment (En)	Increased total phenolic content and AOC; Higher group B vitamins solubility; Increased SCFAs (acetic acid and propionic acid) content;	[23,24]
SSF assisted by En	Increased total phenolic content and AOC.	[25]

Abbreviations: SCFA, short chain fatty acids; SSF, solid state fermentation; AOC, antioxidant capacity; WHC, water holding capacity; OHC, oil holding capacity; WS, water solubility; CAC, cholesterol absorption capacity.

**Table 3:** Thermal and physical treatments applied to rice bran by-product and corresponding outcomes.

Treatment Used	Outcomes	References
Hot-air and FIR	Higher content of bioactive compounds (tocopherols and phenolic acids); Higher AOC.	[30]
Extrusion	Increased arabinoxylans extraction as screw speed increase; Improvement of the emulsifying and foaming properties.	[31,32]
Extrusion plus enzyme	Improved dietary fibres solubility;	[26]
Particle size reduction (dry fractionation and micronization)	Dietary fibres enrichment with modified functional properties; 1.5–5-fold increment of saturated mono- and di-acylglycerols with potential emulsifying properties in fine RB fraction. Increased protein content and solubility; Increased colloidal stability;	[34,35,37]
High Hydrostatic Pressure (HHP)	Increased ferulic acid extractability; Inhibition of linoleic acid oxidation; Higher WBC, OBC, emulsifying stability, improved protein surface hydrophobicity, foaming creation and stabilization.	[27,38]

Abbreviations: RB, rice bran; FIR, far-infrared radiation; WBC, water binding capacity; OBC, oil binding capacity.

**Table 4:** Functional properties of rice bran reported by scientific original papers.

Functional Property	Outcome	References
Water absorption capacity	Relevant water retention (>3 g water/g);	[19,37,38,83,84]
Oil binding capacity	High oil retention due to insoluble dietary fibres (>3 g oil/g);	
Jellification	Jellying of hot water solution at 10–18% RB concentration;	
Emulsion stability	Formation (41–57%) and stabilization of oil in water emulsion (39–74%);	
Foaming stabilization	Low foam formation, but conjointly to other foaming agents is a good stabilizer;	
Protein solubility	High solubility (around 50%) at acidic (3) and basic (8) pH.	
